# Toward a common interpretation of the 3Rs principles in animal research

**DOI:** 10.1038/s41684-024-01476-2

**Published:** 2024-11-15

**Authors:** Jan Lauwereyns, Jeffrey Bajramovic, Bettina Bert, Samuel Camenzind, Joery De Kock, Alisa Elezović, Sevilay Erden, Fernando Gonzalez-Uarquin, Yesim Isil Ulman, Orsolya Ivett Hoffmann, Maria Kitsara, Nikolaos Kostomitsopoulos, Winfried Neuhaus, Benoit Petit-Demouliere, Simone Pollo, Brígida Riso, Sophie Schober, Athanassia Sotiropoulos, Aurélie Thomas, Augusto Vitale, Doris Wilflingseder, Arti Ahluwalia

**Affiliations:** 1 Faculty of Arts and Science, Kyushu University, Fukuoka, Japan; 2 3Rs Centre Utrecht, Utrecht University, Utrecht, The Netherlands; 3 German Federal Institute for Risk Assessment (BfR), German Centre for the Protection of Laboratory Animals (Bf3R), Berlin, Germany; 4 Department of Philosophy, University of Vienna, Vienna, Austria; 5 Vrije Universiteit Brussel, Innovation Centre-3R Alternatives (IC-3Rs), Brussels, Belgium; 6 University of Sarajevo - Faculty of Pharmacy, Sarajevo, Bosnia and Herzegovina; 7 Faculty of Health Science, Nursing Department, Cukurova University, Adana, Turkey; 8 TARCforce3R, University Medical Center Mainz, Mainz, Germany; 9 Acibadem University School of Medicine, History of Medicine and Ethics, Istanbul, Turkey; 10 Department of Animal Biotechnology, Institute of Genetics and Biotechnology, Hungarian University of Agriculture and Life Sciences, Gödöllő, Hungary; 11 BIOEMTECH, Athens, Greece; 12 Biomedical Research Foundation of the Academy of Athens, Athens, Greece; 13 AIT - Austrian Institute of Technology, Vienna, Austria; 14 PHENOMIN-ICS, CNRS, INSERM, Université de Strasbourg, Strasbourg, France; 15 Dipartimento di Filosofia, Università di Roma Sapienza, Rome, Italy; 16 Faculdade de Medicina, Universidade de Lisboa, Lisbon, Portugal; 17 Institute of Science and Technology Austria (ISTA), Klosterneuburg, Austria; 18 French 3R Center GIS FC3R, Maisons-Alfort, France; 19 Animal Sciences & Technology, Clinical Pharmacology and Safety Sciences, R&D, AstraZeneca, Cambridge, United Kingdom; 20 Center for Behavioural Science and Mental Health, Istituto Superiore di Sanità, Rome, Italy; 21 Ignaz Semmelweis Institute, Interuniversity Institute for Infection Research, Infectiology & Virology Unit, Vetmeduni Vienna, Austria; 22 Centro 3R, Department of Information Engineering, University of Pisa, Pisa, Italy

**Keywords:** Ethics, Lab life

## Abstract

Many scientific breakthroughs have depended on animal research, yet the ethical concerns surrounding the use of animals in experimentation have long prompted discussions about humane treatment and responsible scientific practice. First articulated by Russell and Burch, the 3Rs Principles of Replacement, Reduction, and Refinement have gained widespread recognition as basic guidelines for animal research. Over time, the 3Rs have transcended the research community, influencing policy decisions, animal welfare advocacy and public perception of animal experimentation. Despite their broad acceptance, interpretations of the 3Rs vary substantially, shaping statutory frameworks at various levels, with both technical and practical impacts.

A notable moment for the recognition of the 3Rs was the adoption of the European Union Directive 2010/63/EU “on the protection of animals used for scientific purposes^1^”. This recognition not only led to the establishment of national committees and animal welfare bodies charged with monitoring and facilitating research practices, but also to a marked increase in 3Rs initiatives across Europe, including the formation of 3Rs centers and platforms^2^. The 3Rs now elicit a level of recognition across the globe that makes them a powerful framework even with respect to the use of animals in food production and wildlife management^3,4^. The expansion in the application of the 3Rs is also coherent with the One Health concept, defined by the World Health Organization as “an integrated, unifying approach that aims to sustainably balance and optimize the health of people, animals and ecosystems”. For example, it has been suggested that organoids derived from a variety of species could be employed to study diseases that affect both humans and animals — such as different forms of cancer or host-pathogen interactions and zoonoses — putting health and safety in a broader context compared, respectively, to investigations on single aspects of oncological diseases or zoonotic pathogens in a limited number of laboratory animal species^5^. In this direction, the theme of the next World Congress on Alternatives and Animal Use in the Life Sciences (WC13, in Brazil): “3Rs Integrating 3 Worlds: Human, Animal and Environmental Health” indicates recognition of a link between One Health and the 3Rs and their collective impact on advancing health outcomes across interconnected systems.

At this critical moment of broad recognition of the 3Rs, it is essential to work from a shared understanding of their meaning among the scientific community and stakeholders to facilitate their wider implementation, foster progress and innovation in research, target funding, and streamline their branding to the public. Indeed, the different ways of interpreting and implementing the 3Rs can sometimes lead to misunderstandings of the efforts made by the scientific community in biomedical research. A common starting point to harmonize their meaning is also desirable for comparison and consistency across jurisdictions^6^. Important points of discussion are the function of the 3Rs, the animals under consideration, the range of replacement, the proper targets for reduction, the refinement goals, and the order and priority of the 3Rs. Some researchers have even suggested adding extra Rs to the original concept of the 3Rs to compensate for the perceived omissions relating to good research practices, social values and scientific integrity. The two most-cited extra Rs are Responsibility and Reproducibility, and the current record sits at 12 Rs^7^.

To address these issues, we formed a working group within the COST Action IMPROVE CA21139 *“*Using the 3Rs concepts to improve the quality of biomedical science*”*, to establish whether we could obtain a shared interpretation within our network of 3Rs professionals.

The working group, composed of biomedical researchers, veterinarians and bioethicists, prepared the present proposal for a common interpretation, which was then sent to the members of the network, asking if they agreed with it. The majority of members of the COST Action IMPROVE approved the interpretation presented here. We carefully review Russell and Burch’s original definitions, clarifying them and seeking to render them consistent with our current understanding of sentience and pain in animals, while considering both the legal framework provided by Directive 2010/63/EU and the updated interpretations proposed by the U.K. National Centre for the 3Rs (NC3Rs).

## The original definitions

Russell and Burch first defined the 3Rs in Chapter 4 (“The Removal of Inhumanity”) of their landmark publication “The Principles of Humane Experimental Technique^8^”: *“*Replacement means the substitution for conscious living higher animals of insentient material. Reduction means reduction in the numbers of animals used to obtain information of a given amount and precision. Refinement means any decrease in the incidence or severity of inhumane procedures applied to those animals which still have to be used”.

They made a further distinction with respect to Replacement in Chapter 5 (“Modes of Absolute and Relative Replacement”). “In relative replacement, animals are still required, though in actual experiment, they are exposed, probably or certainly, to no distress at all. In absolute replacement, animals are not required at all at any stage.”

## The function of the 3Rs

Although Russell and Burch intended their work to “serve as an initial source and guide for studies in this field” (Chapter 1, “Scope of the Study”), with their emphasis on “the removal of inhumanity”, they presented the design of the 3Rs Principles as a project in which animal research converges with ethics toward medical or scientific progress. Indeed, they noted that “we owe to animal experimentation many if not most of the benefits of modern medicine and countless advances in fundamental scientific knowledge”. In the same breath, they claimed that “by now it is widely recognized that the humanest possible treatment of experimental animals, far from being an obstacle, is actually a prerequisite for successful animal experiments”. In line with this notion of convergence between good science and ethics, the 3Rs Principles are widely considered as both a guidance toward best practices as well as an ethical framework from which to take a more holistic and responsible approach towards animal research^9^. A common interpretation of the 3Rs Principles can thus be regarded as the outcome of an ongoing cultural debate on the moral status of animals and its impact on the ethics and practice of laboratory animal research. Only through discussion can we advance animal welfare and make scientific progress, fine-tuning shared interpretations and definitions as we move forward.

Thus, we regard the 3Rs not merely as a technical tool or checklist for scientists or ethics committees to judge the necessity of a proposed animal experiment. Instead, we emphasize the dynamic nature of good research practices — in pace with scientific progress — and envisage the 3Rs as a broader, evolving framework promoting continued improvement of scientific outcomes and animal welfare. While there are many variations in the practice as well as the legislation and monitoring of animal research across the globe, a common understanding of the 3Rs should gradually lead to greater alignment across jurisdictions, in as far as scientific and medical progress as well as human, animal and ecosystem welfare represent universal goals.

## The animals under consideration

Russell and Burch’s reference to “conscious living higher animals” likely reflects the level of understanding of sentience at the time and is too vague to offer guidance. Directive 2010/63/EU does not define the term animal (hence its ‘ordinary’ or ‘plain’ meaning is implicit), but states that the protection of animals used for scientific purposes applies to non-human vertebrates and cephalopods, given the scientific evidence of their ability to “experience pain, suffering, distress and lasting harm” (Recital 8 and Article 1(3)). It further specifies the life stages the protection covers: “live” specimens, beginning with independently feeding larval forms and fetal forms of mammals from the last third of their normal development (Recital 9). According to the NC3Rs, under the UK’s Animals Act 1986, cephalopods are protected from the moment they hatch.

We note that since 2022, vertebrates, cephalopods and decapods have been recognized as sentient beings under the UK’s Animal Welfare (Sentience) Act. More recently, a group of scientists has declared that there is sufficient evidence to support sentience in both vertebrates and invertebrates^10^. Given that the attribution of sentience may also be expanded to other species according to new scientific and animal welfare knowledge, we refer here to all animals.

## Replacement

Directive 2010/63/EU requires Member States to *“*ensure that, wherever possible, a scientifically satisfactory method or testing strategy, not entailing the use of live animals, shall be used instead of a procedure” (Article 4(1)). NC3Rs is more explicit in this regard, defining Replacement as “Accelerating the development and use of predictive and robust models and tools, based on the latest science and technologies, to address important scientific questions without the use of animals*”*. Thus, both the Directive and NC3Rs make room for non-animal based new technologies under the principle of Replacement. The new methods, also referred to as NAMs (New Approach Methodologies), represent cutting-edge scientific tools that were likely inconceivable when the 3Rs were first articulated 65 years ago. NAMs do not necessarily provide a one-to-one substitute for traditional animal-based techniques, but they offer novel ways to address research questions that animal models might not be able to answer.

We therefore argue that the concept of Replacement could be expanded and updated to include the development of these rapidly emerging non-animal approaches. Thus, NAMs can act as specific replacements (surrogates for existing animal tests) or as proactive replacements, opening new and potentially unprecedented avenues of research without relying on animals. Hence, the concept of Replacement is not limited to the substitution of a procedure X by a procedure Y, which avoids the use of animals and that would produce the same piece of information (such as using in vitro epidermal models derived from human skin to assess the skin irritation potential of chemicals); it should also be seen as an effort to conduct research to obtain new information without the use of animals, particularly in fields such as neuroscience and toxicology that have traditionally relied heavily on them.

We note that NC3Rs makes a distinction between “Full Replacement” and “Partial Replacement” that echoes Russell and Burch’s separation of Absolute and Relative Replacement. Directive 2010/63/EU does not make such a distinction. We choose to avoid the dichotomy as it could dilute the concept of Replacement and create confusion with the concept of Refinement (as detailed below), preferring Russell and Burch’s recommendation of Absolute Replacement as “the absolute ideal”. Thus, we consider Replacement to be a question of whether animals are being used in any way, including, for instance, the killing of animals to harvest organs or tissues or the use of animal-derived products such as sera, antibodies or extracellular matrices in biomedical applications. In applications where animal health is the objective (e.g., animal vaccine development or ecotoxicology), researchers should, as far as possible, strive to use new methods akin to those being developed in biomedicine (e.g., induced pluripotent stem cells, organoids, recombinant proteins).

Although the absolute ideal of completely avoiding the use of animals is currently unattainable, it serves as a guiding vision. Scientific transparency can be maintained with the public and funders by acknowledging our current limitations and by articulating commitments to Replacement, leveraging innovative approaches, and fostering collaboration within the scientific community.

Our interpretation of Replacement is: Replacement means to conduct research that completely avoids the use of animals in scientific investigation, regulatory testing, and education.

## Reduction

Directive 2010/63/EU briefly touches on the concept of Reduction as the minimization of animal numbers without compromising scientific objectives to provide reliable results (Recital 13 and Article 4(2)). Similarly, NC3Rs states that Reduction means “Appropriately designed and analyzed animal experiments that are robust and reproducible, and truly add to the knowledge base”. In doing so, both allude to the need for good research practices.

This implies a shift of agency as compared to Russell and Burch; instead of principal investigators making decisions about the minimal number of animals needed for a given research project, Reduction is interpreted as a challenge for the entire community of stakeholders. Thus, to achieve a proper reduction of the number of animals being used in research — while maintaining or even improving knowledge outcomes or using the same number of animals to generate more data and knowledge^11^ — efforts should be coordinated across legislators, regulatory organizations, institutions, research groups, as well as individuals.

In practice, the aim of coordination is to maximize efficiency in data collection, using fewer animals through better orchestrated research across labs and research communities. While it obviously remains the responsibility of every Principal Investigator to design and implement their research based on a proper sample size calculation, the principle of Reduction further encourages collaboration, data sharing and pooling of resources. Thus, we advocate for a dynamic, forward-looking application of Reduction, aiming at the improvement of research practices leveraging on scientific and technological progress. The application of Reduction should not only be seen as a matter of policing efficiency in terms of sample size calculation in individual laboratories, but also as a matter of innovation in collaborative research practices that can achieve better and more impactful results while using fewer animals.

Our interpretation of Reduction is: Reduction means to minimize the number of animals necessary to provide reliable and useful information in scientific investigation, regulatory testing, and education.

## Refinement

Both Directive 2010/63/EU and NC3Rs state that Refinement applies to all aspects of animal use, from breeding and accommodation to the experimental procedures and killing, reflecting Russell and Burch’s original conceptualization. To use the language of Directive 2010/63/EU, the principle is to minimize any “pain, suffering, distress, or lasting harm”. With ‘minimize,’ we refer to the “least possible amount,” which includes complete elimination or a reduction of any pain, suffering, distress or lasting harm, without compromising scientific gain.

Refinement is typically perceived as an indirect aspect of generating data — not the data collection itself, but the required preparations and animal care — for example, optimal husbandry or less invasive ways of administering substances. However, the design of any new testing or research method that aims to minimize animal suffering or distress represents an effort of Refinement. This can also be achieved by switching to a species that is less demanding in terms of welfare needs or through the design and choice of scientific and humane endpoints (e.g., ending a study earlier, without compromising information output).

While switches of animal models or modifications in the animal paradigm have sometimes been labeled ‘Relative’ or ‘Partial’ Replacement, we consider them as forms of Refinement. This is in line with Russell and Burch’s original proposition, and tallies with Recital 13 and Article 4(3) of Directive 2010/63/EU. Refinement thus ranges from, say, creating enriched environments in which animals can display more natural behavior to using zebrafish rather than mice to study alcohol-induced locomotor aberrations^12^. We should note that while zebrafish are often considered ‘lower’ on the evolutionary scale than mice, our present level of knowledge on sentience across species is still limited. Nonetheless, zebrafish are more amenable to high-throughput analyses and have faster generation times, potentially providing more information in less time. Ultimately, as with the efforts towards Reduction, the choice of species and study design should be based on a shared concern for good practices.

Our interpretation of Refinement is: Refinement means any measure to improve the welfare or minimize the pain, suffering, distress or lasting harm of animals used in scientific investigation, regulatory testing and education.

## The priority and sequence among the 3Rs

Russell and Burch intended a clear order among the 3Rs, with Replacement as the preferable and first principle, that “is always a satisfactory answer” (Chapter 4), followed by Reduction and then Refinement. Although this order has sometimes been ignored, or even reversed^13^ we reaffirm that the logic of minimizing animal suffering dictates that researchers should first and foremost aim for Replacement (also reflected in Directive 2010/63/EU’s Recital 11).

**Fig. 1:**
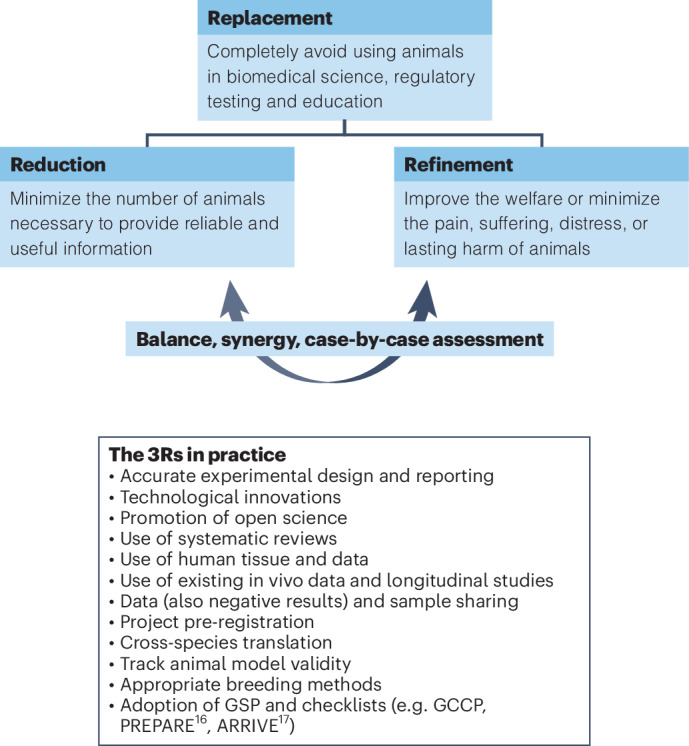
A common interpretation of the 3Rs and their hierarchy. The lower panel lists some of the practices that can be implemented to promote the 3Rs and thus animal welfare and good science. GSP – Good Scientific Practice, GCCP – Good Cell Culture Practice. PREPARE – Planning Research and Experimental Procedures on Animals: Recommendations for Excellence^16^ and ARRIVE – Animal Research: Reporting of In Vivo Experiments^17^ are guidelines for planning and reporting animal experiments.

When Replacement is not an option, researchers should proceed with applying Reduction and Refinement in a balanced, synergistic manner. Balancing Reduction and Refinement, for instance, when using fewer animals but at greater cost to their welfare^14^ or using a larger number of fish instead of fewer mice, requires a case-by-case holistic assessment of projects, considering both validity and harm-benefit analyses to manage potential conflicts^15^. The successful integration of the 3Rs requires careful planning, execution, and support across the entire stakeholder chain. Figure 1 summarizes the interpretations and lists some of the practices by which researchers can implement the 3Rs Principles.

In conclusion, we propose a concise, updated interpretation of the 3Rs that takes into account our current understanding of sentience and pain in animals and is in alignment with the basic philosophy of Russell and Burch. As argued here, a common understanding is pivotal to harmonized implementation of the 3Rs, engaging diverse stakeholders and underscoring the importance of robust scientific practices. Applying the 3Rs Principles then becomes a pathway to achieving superior research outcomes and advancing scientific knowledge while taking care of animals in the best way possible.
